# Tracing histoplasmosis genomic epidemiology and species occurrence across the USA

**DOI:** 10.1080/22221751.2024.2315960

**Published:** 2024-03-11

**Authors:** Bernardo Guerra Tenório, Daniel R. Kollath, Lalitha Gade, Anastasia P. Litvintseva, Tom Chiller, Jeff S. Jenness, Jason E. Stajich, Daniel R. Matute, Andrew S. Hanzlicek, Bridget M. Barker, Marcus de Melo Teixeira

**Affiliations:** a Faculty of Medicine, University of Brasília, Brasília, Brazil; b Pathogen and Microbiome Institute, Northern Arizona University, Flagstaff, AZ, USA; c Mycotic Diseases Branch, Centers for Disease Control and Prevention, Atlanta, GA, USA; d School of Forestry, Northern Arizona University, Flagstaff, AZ, USA; e Department of Microbiology & Plant Pathology and Institute for Integrative Genome Biology, University of California, Riverside, CA, USA; f Biology Department, University of North Carolina, Chapel Hill, NC, USA; g MiraVista Diagnostics, Indianapolis, IN, USA; h Department of Veterinary Clinical Sciences, Oklahoma State University, Stillwater, OK, USA

**Keywords:** Histoplasmosis, molecular epidemiology, *Histoplasma ohiense*, *Histoplasma mississippiense*, genomics, species distribution modelling

## Abstract

Histoplasmosis is an endemic mycosis in North America frequently reported along the Ohio and Mississippi River Valleys, although autochthonous cases occur in non-endemic areas. In the United States, the disease is provoked by two genetically distinct clades of *Histoplasma capsulatum sensu lato*, *Histoplasma mississippiense* (Nam1) and *H. ohiense* (Nam2). To bridge the molecular epidemiological gap, we genotyped 93 *Histoplasma* isolates (62 novel genomes) including clinical, environmental, and veterinarian samples from a broader geographical range by whole-genome sequencing, followed by evolutionary and species niche modelling analyses. We show that histoplasmosis is caused by two major lineages, *H. ohiense* and *H. mississippiense*; with sporadic cases caused by *H. suramericanum* in California and Texas. While *H. ohiense* is prevalent in eastern states, *H. mississipiense* was found to be prevalent in the central and western portions of the United States, but also geographically overlapping in some areas suggesting that these species might co-occur. Species Niche Modelling revealed that *H. ohiense* thrives in places with warmer and drier conditions, while *H. mississippiense* is endemic to areas with cooler temperatures and more precipitation. In addition, we predicted multiple areas of secondary contact zones where the two species co-occur, potentially facilitating gene exchange and hybridization. This study provides the most comprehensive understanding of the genomic epidemiology of histoplasmosis in the USA and lays a blueprint for the study of invasive fungal diseases.

## Introduction

Histoplasmosis is an endemic mycosis that is distributed in specific tropical and subtropical areas of the globe with high prevalence in the eastern portion of the United States, particularly in the Mississippi and Ohio River Valleys. The disease is caused by the dimorphic fungus *Histoplasma* sp*.* and is reported in all continents including its recent discovery in Antarctica [1,2]. The filamentous form of the fungus is frequently associated with soils enriched with bat guano or bird droppings, with average temperatures of 25°C, low luminosity, and high humidity [3–5]. During this phase, micro- and macroconidia are produced, and the infection is triggered when susceptible hosts inhale these aerosolized particles upon soil disturbance reaching the lungs’ alveoli. The fungus transitions to yeast form at 37°C and specific metabolic conditions imposed by the host [6]. Cases and outbreaks have been linked to construction sites, abandoned buildings, caves, and chicken coops [7]. Histoplasmosis is usually asymptomatic or develops clinical symptoms similar to other acute pulmonary infections such as fever, cough, and fatigue [2]. Progressive and disseminated histoplasmosis is commonly observed in immunocompromised patients and is often fatal, especially in HIV + patients [8].

Histoplasmosis is highly endemic in the United States and up to 80,000 documented cases were reported between 2006 and 2017. In 2019, there were 1124 confirmed cases, and it is estimated that approximately 500,000 natural *Histoplasma* infections occur annually within the United States and about 1% are symptomatic [9–11]. Large-scale histoplasmin skin test surveys revealed a high prevalence of *Histoplasma* infection around the Ohio and Mississippi Valleys, suggesting that 60% to 90% of those residents have been exposed to the fungus [12,13]. At least 105 histoplasmosis outbreaks were documented between 1938 and 2013 in the USA, affecting 2850 individuals [7]. The epidemiological scenario has been changing since autochthonous cases have been reported outside the proposed endemic zone in the US. The disease has been observed in the western portion of the United States, extending to Canada, and reported at a lower incidence in southern and central regions of the United States [12, 13]. However, the case rates may be significantly underestimated due to a lack of surveillance. The disease has a mandatory notifiable status in thirteen of the fifty USA states [11, 14]. In the USA and Canada, histoplasmosis is caused by *H. mississippiense* (NAm1) and *H. ohiense* (NAm2), while in Latin America, the disease is caused by *H. suramericanum* (LAmA), *H. capsulatum sensu stricto* (Panama) and other cryptic genotypes [15–17]. In recent studies, Teixeira *et al*., 2016 also observed that cat-and-bat-associated genetic clusters are observed in both Latin and North American *Histoplasma* species, suggesting that alternate hosts may play an important role in species diversification [15]. The phylogenetic species concept [18] strongly advocates for the recognition of multiple species boundaries within *Histoplasma* [15,16,19,20,21]*.* According to electrophoresis karyotype and genome sequencing analysis, the *Histoplasma* spp. genomes have 5–7 chromosomes spanning 30.4 Mb up to 39.4Mb and such variation is dictated by different genotypes and geographic origin [22,23].

Molecular epidemiological studies of histoplasmosis have been scarce in the USA, even though the disease is prevalent and emergent. To date, all the molecular-based epidemiological studies conducted in the USA (*i*) have used only a few genetic markers, or (*ii*) were obtained only from patients in Missouri and Louisiana, which represents only a portion of the potential range of *Histoplasma* [15,16,19]. In this study*,* we bridge these gaps by studying 93 *Histoplasma* genomes obtained from a broader geographic range. These isolates were obtained from clinical, veterinary, and environmental sources, which were collected in 19 US states to classify the aetiological agents of American histoplasmosis and species distribution in a broader context. By using data from whole-genome sequence typing as a tool for molecular epidemiology, we argue it is possible to obtain a better understanding of *Histoplasma*’s geographic range and pinpoint which *Histoplasma* genotypes are more likely to be found in each region of the country. Furthermore, this framework allows seeking for clinically-relevant phenotypes between species causing histoplasmosis in the American continent.

## Material and methods

### Fungal strains, DNA extraction and genome sequencing

Sixty-two environmental, clinical, and veterinary isolates from this study, were received in the Mycotic Diseases Branch Laboratory at the Centers for Disease Control and Prevention for routine fungal identification or as part of ongoing fungal disease surveillance. Upon arrival, isolates were identified by sequencing of the ITS2 region of the rDNA and were stored in 20% glycerol at −70°C. Isolates were grown on brain heart infusion (BHI) agar at 25°C not more than 10 days. Genomic DNA was extracted by using the DNeasy Blood and Tissue kit (Qiagen, Gaithersburg, MD) according to the manufacturer’s recommendations and verified using 0.8% gel electrophoresis. The quantification estimate and purity was measured on a NanoDrop™ 2000c spectrophotometer. All procedures were conducted in a BSL3 laboratory due to the high fungal infectivity of *Histoplasma*’s mycelial phase. All paired-end sequencing libraries were prepped using the Kapa Biosystems kit (Kapa Biosystems, Woburn, MA) and approximately 1ug of DNA was used as input material. DNA sequencing was performed on an Illumina HiSeq 2500 (100 bp, high output v3 or v4 chemistries) or in an Illumina NextSeq 550 equipment (150bp, high output v2).

### SNP calling, phylogeny and population structure

Paired-end reads were trimmed using Trimmomatic V0.32 [24] using the following parameters: SLIDINGWINDOW:10:30, LEADING:28, TRAILING:28 and MINLEN:80 and aligned to the *H. mississippiense* NAm1 reference strain (AAJI00000000.1) using the Burrows–Wheeler Aligner (BWA – v 0.7.17 – [25]). Genomic coverage was assessed using the tinycov script (https://github.com/cmdoret/tinycov). Read files from each strain were remapped to its correspondent.bam file to identify INDELS and purge unmapped DNA segments utilizing the RealignerTargetCreator and IndelRealigner programs (GATK toolkit v3.3-0 [26]). UnifiedGenotyper was used to identify the SNPs and hard filters were applied for the .vcf files using the following parameters: QD = 2.0, FS_filter = 60.0, MQ_filter = 30.0, MQ_Rank_Sum_filter = -12.5, Read_Pos_Rank_Sum_filter = −8. SNP’s with n low coverage (<10X) or low variant allele calls (<90%) or falling within duplicated regions (detected by NUCmer tool [27]) in the reference genome were purged from the final dataset. The whole-genome SNP matrix composed of 93 isolates was used for evolutionary analysis. Maximum Likelihood (ML) phylogenetic trees were built using IQ-TREE v2.1.1 [28]. Branch support was determined using bootstrap and SH-like approximate-likelihood ratio tests [29, 30]. Population composition was calculated using two approaches: (i) fastSTRUCTURE [31] was used to investigate the number of cryptic populations within *H. mississippiense* + *H. ohiense* or in *H. mississippiense* and *H. ohiense* separately. The best scenario (k) of number of populations was inferred by the maximization of log-likelihood scores simulated using the choosek.py function. (ii) Principal Coordinate Analysis (PCA) implemented in the R package Adegenet v1.3 [32] was used to evaluate the population distribution of the same datasets based on the genetic variation distributed in two coordinates, namely PC1 and PC2. Finally, we studied the amount of genetic diversity in *H. mississippiense* and *H. ohiense* by calculating the average nucleotide diversity (π) within species using the Python script *popgenWindows.py* available at https://github.com/simonhmartin/genomics_general. The mating-type idiomorph of each isolate was determined by aligning either the *MAT1-1* (EF433757) or *MAT1-2* (EF433756) loci to each corresponding *Histoplasma* genome. The Chi-squared test was employed to assess whether there was a deviation from the expected 50:50 mating-type distribution.

### Species distribution modelling

Geographic coordinates from clinical, veterinary and environmental samples from the sequenced genomes were retrieved from records available at the US-CDC and from Sepulveda et al., [16]. To improve the robustness of the niche modelling, we included samples that were genotyped using single or multiple loci as published elsewhere [19,33,34], totalizing 152 entries. Samples with city-level metadata (*n* = 29) were included as high confidence geographical locations. Samples containing state-level information (*n* = 123) were randomized within that given state using Google Earth Engine [35] through the randomPoints function. Samples without any geographical information were excluded for niche modelling analysis. Climate variables used to predict the distribution of the two *Histoplasma* species were obtained from the WorldClim 2 database [36]. These data represent 1970–2000 averages of climatic variables derived from ground-based meteorological measurements. These variables encompass temperature, precipitation, and other ecologically significant environmental conditions for geographic niche occupation.

To predict the distribution of the two *Histoplasma* species, separate species distribution models (SDM) were calculated based on the Maximum Entropy (MaxEnt) algorithm [37]. The MaxEnt models are based on presence-only geographic data. To account for absences and areas where presences are not accounted for but may be possible, we added a random sampling of 1000 background points to both models. The models were constructed and evaluated using the dismo R package version 1.3–14 [38]. Separate MaxEnt models were run for the distribution of *H. ohiense* and *H. mississippiense*. In both models, climate variables were assessed for collinearity with a pairwise Spearman’s correlation test with a threshold of 0.7, followed by leave-one-out Jackknife test among all correlated variables. Model performance was assessed using the area under the curve (AUC) of the receiver operating characteristic (ROC). To detect areas of niche overlap between the two species of *Histoplasma*, the predicted distributions were overlaid onto a map. A threshold of predicted probability of 0.75 was set to examine areas that have highest probability of high-confidence overlap.

## Results

### Phylogenetic background of *Histoplasma* strains from the US

We sequenced and deposited 62 *Histoplasma* isolates, with coverages ranging from 15.82X to 102.7X, from 19 U.S. states in the Sequence Read Archive (SRA) under accession numbers (SRR25580010- SRR25580071). A total of 1,360,150 SNPs, covering 903,017 informative sites, were analyzed across 93 genomes, which included the 62 novel isolates, 30 from a previous study [16], and the reference genome NAm1. Notably, Missouri and Georgia had the highest numbers of human isolates (*n* = 17 and *n* = 11, respectively), Indiana had the most environmental isolates (*n* = 8), and Oklahoma recorded a substantial number of pet-derived isolates (*n* = 15); see Supplementary Table 1.

Our study revealed that the majority of U.S. isolates belonged to two *Histoplasma* species: *H. mississippiense* (NAm1) and *H. ohiense* (NAm2). Soil-derived strains from both species were identified in Indiana, suggesting the potential for soil-borne infections by these genotypes in the state. Within *H. ohiense,* human-derived strains constituted 65% of the isolates, followed by 21% of environmental isolates, 6% from wild mammals, and 4% from veterinary sources, with 4% having no specified source records. These isolates were distributed across 17 states. In contrast, within *H. mississippiense*, we observed two monophyletic clades, named Clade I and Clade II. Clade I primarily consisted of cat (47.4%) and dog-derived (26.3%) strains, all from Oklahoma, with human-derived (21%) strains in California, Colorado, and Kansas. Environmental strains (5.3%) were confined to Indiana within *H. mississippiense*. Clade II comprised solely of human-derived strains distributed across Arkansas, Indiana, and Missouri. Additionally, two strains nested within *H. suramericanum* were identified, one from Texas and another from Richmond, California, indicating potential endemicity in the Southern and Western USA. Both cases had unknown travel history.

**Figure 1.**
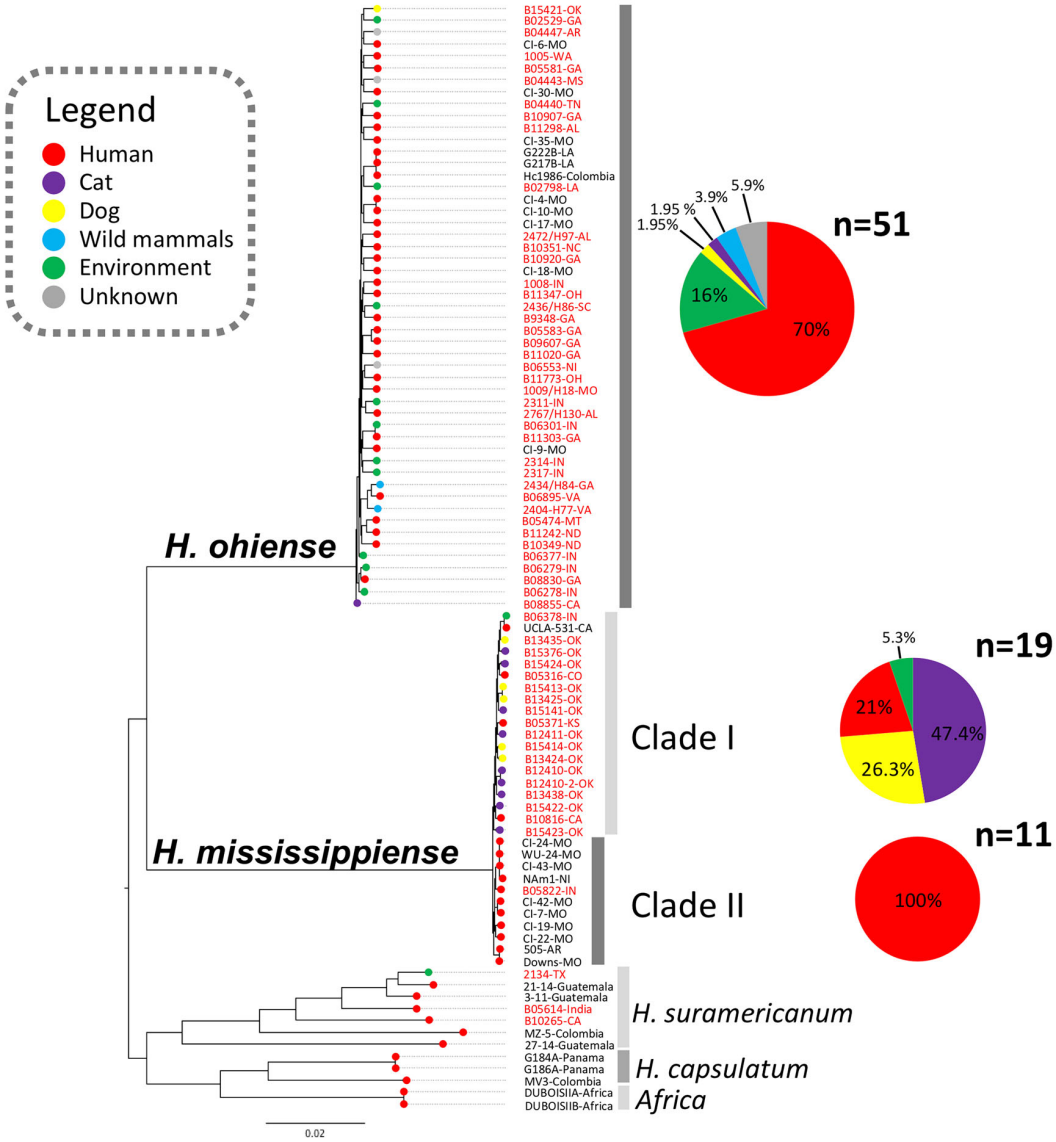
Whole-genome phylogenetic tree of *Histoplasma* isolates collected in the United States. The branches are proportional to the number of mutations and 1000 ultrafast bootstraps and SH-aLRT were used supporting the major branches of the tree. The nodes in the tree represent common ancestors, and the branching points indicate the divergence of lineages. The tips represent each *Histoplasma* isolate and are colour-coded according to its source; the percentage of each source of isolation was plotted in a pie chart. The state or country of isolation were added next to each taxa; two isolates had no information regarding the place of isolation (NI).

**Figure 2.**
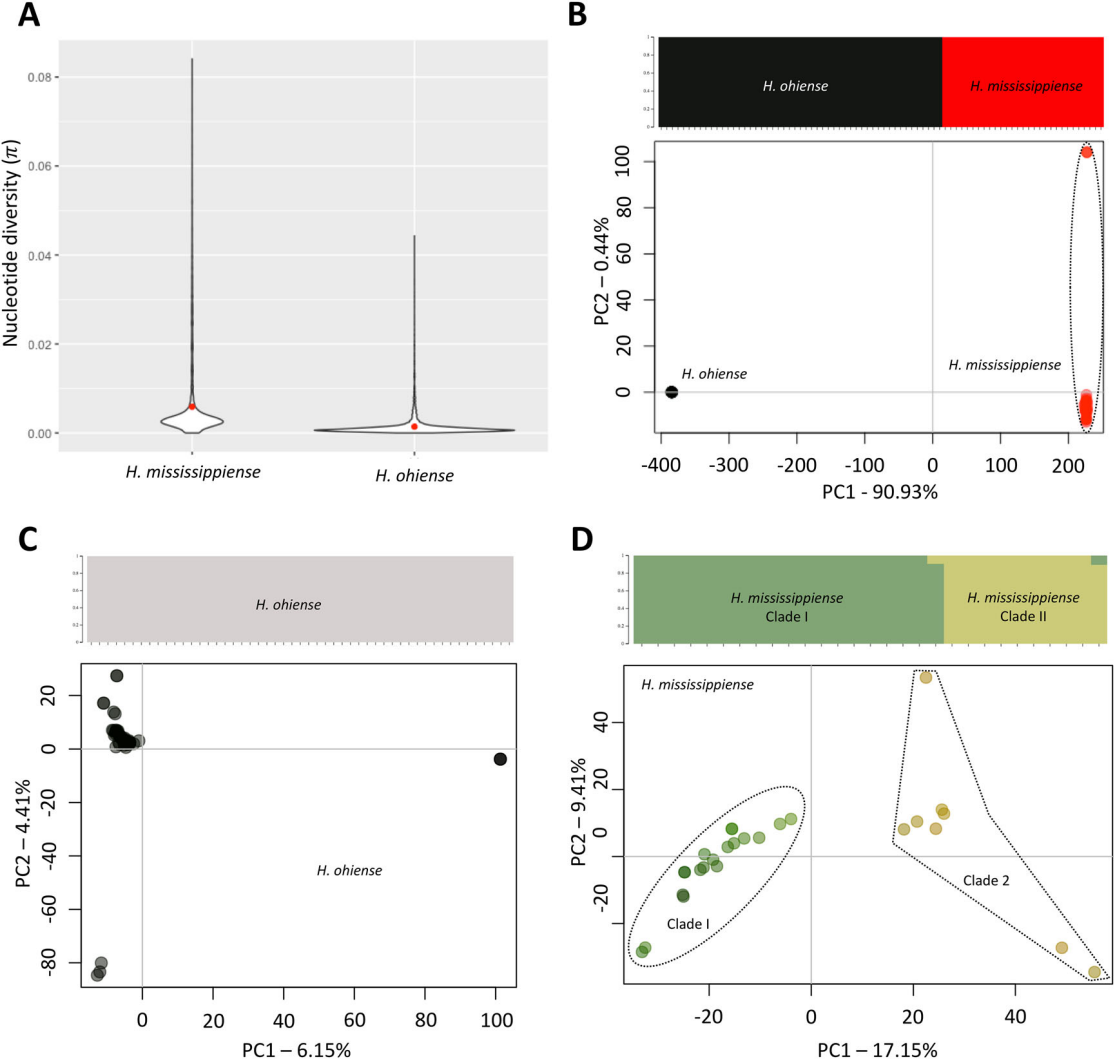
Diversity analysis and population genetic analysis of *Histoplasma* sp. in the USA. (A) Violin plot explaining the genome-wide dispersion of nucleotide diversity in *Histoplasma ohiense* and *H. mississippiense* (π). Red dots represent the average π values within each species. Structure plots and Principal Coordinate Analysis (PCA) suggests different patterns of population structure in *Histoplasma ohiense* and *H. mississippiense* together (B) or *H. ohiense* (C) and *H. mississippiense* (D) separately. Structure analysis revealed the presence or absence of cryptic populations which was inferred by the maximization of log-likelihood scores simulated in the fastSTRUCTURE software and is displayed in the upper panel of each comparisons. Each row represents an individual and the heights and colours of percentage of each population represent the probability of belonging to a given cluster. PCA plots are displayed in the lower panels and depicts the genetic variation distributed in two coordinates, PC1 and PC2, based on polymorphisms and similarities in the genomes of *Histoplasma ohiense* and *H. mississippiense* analyzed together or separately*.*

We next measured the amount of genetic variation within each *Histoplasma* species present in the US. We have found that *H. mississippiense* has a larger nucleotide diversity (π) (π = 0.57%) than *H. ohiense* (π = 0.14%, Figure 2(A)). We further investigated the population structure of both species using the Structure followed by PCA approaches, aiming to identify potentially cryptic populations. Both Structure (upper panel) and PCA (lower panel) that incorporate the two species show, as expected, two distinct and well-defined clusters: *H. mississipiense* and *H. ohiense* (Figure 2(B), Supplementary Figure 1). Most of the observed genetic variation is explained by PC1 (90.93%) which corresponds to the differentiation between *H. mississipiense* and *H. ohiense*. PC2 explain 0.44% of the total variance. Next, we studied the extent of populations within each species. Structure analyses haven’t detected any cryptic population as indicated by Figure 2(C) upper panel and Supplementary Figure 1. Nevertheless, the PCA analysis in Figure 2(C) (lower panel) did identify three subtle clusters. It is important to note that PC1 accounts for only 6.15% of the variance, and PC2 for 4.41%. In contrast, structure analysis did find two discrete populations within *H. mississippiense*, which corresponds to *H. mississipiense* clades I and II (Figure 2(D) upper panel, Supplementary Figure 1), in agreement with phylogenomic analysis (Figure 1). The PCA plots do also discriminate these two populations along the PC1 axis, which explains 17.15% of the total variation within this species (Figure 2(D), lower panel); PC2 explain 9.41% of the total variance. With respect to the mating-type distributions within *H. mississipiense* and *H. ohiense*, different populations and locations do not differ from the expected 50:50 mating-type distribution in sexual recombining species (*p* > 0.05).

### Geographic distribution and species modelling of *H. mississipiense* and *H. ohiense*

**Figure 3.**
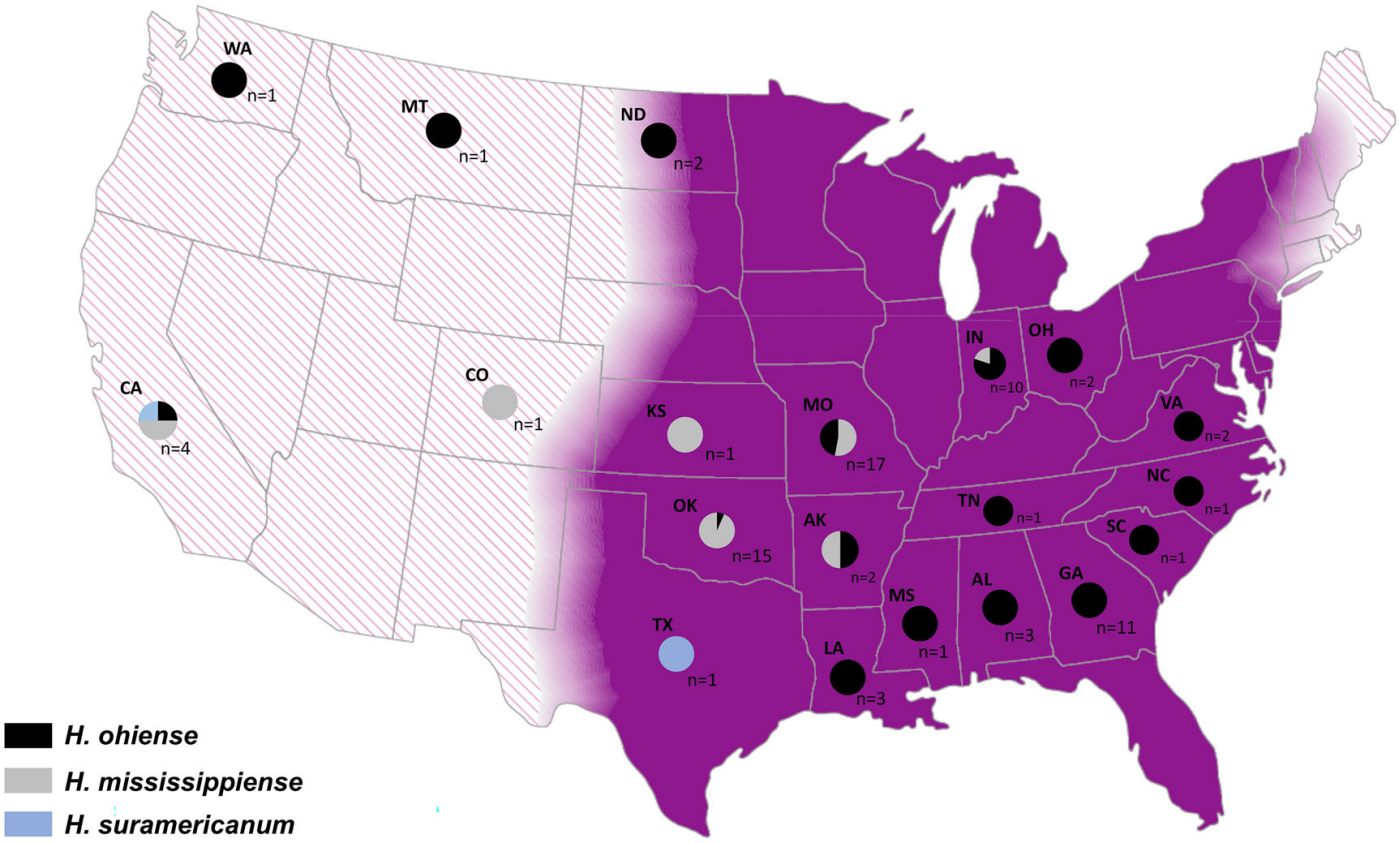
Geographic distribution of *Histoplasma ohiense, H. mississippiense* and *H. suramericanum* in the US territory. Eighty two isolates were genotyped using whole genome phylogenetic typing and were plotted as pie charts to each respective state of origin. *Histoplasma ohiense* is respresented by black while *H. mississippiense* and *H. suramericanum* are represented by grey and blue colours respectively. Two isolates had no information regarding the location of isolation. The background map is coloured in purple representing the known endemic area of histoplasmosis in the mid-eastern of US; hachure area represents potential endemic areas.

**Figure 4.**
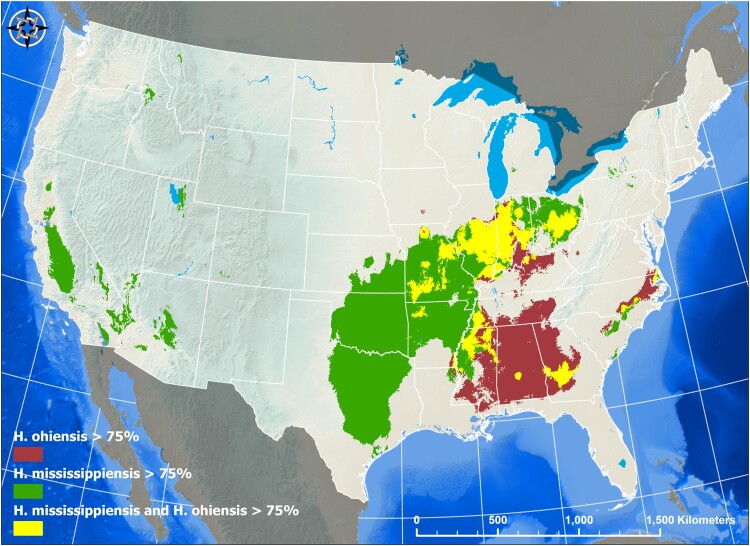
Species Niche Modelling of *Histoplasma ohiense* and *H. mississippiense.* The location of 152 *Histoplasma* typed by whole-genome or other methods were used as presence-only geographic data to generate species distribution models (SDM) based on the Maximum Entropy (MaxEnt) algorithm (25). Separate MaxEnt models were ran for the distribution of *H. ohiense* (red area) and *H. mississippiense* (green area) and performance was assessed using the area under the curve (AUC) of the receiver operating characteristic (ROC). In order to detect areas of niche overlap between the two species of *Histoplasm*a, the predicted distributions were overlayed onto a map (yellow area). A threshold of predicted probability of 0.75 was set to examine areas of that have highest probability of species-specific areas as well as the overlapping areas.

*Histoplasma ohiense* isolates are collected broadly in the USA (17 states) while *H. mississipiense* seems to have a smaller distribution (11 states). While *H. ohiense* is prevalent in eastern portion of the country, *H. mississipiense* was found to be predominant in the central and western portions of the USA (Figure 3). The range of the species does overlap. We found both species in Indiana, Missouri, Oklahoma, and California suggesting that these species might co-occur, which could facilitate gene exchange between species. Notably, both species, *H. mississipiense* and *H. ohiense*, were collected as environmental samples in Indiana (Figure 3). We used species niche modelling to determine if any environmental factors favoured the occurrence of one or both species. The best performing model for *H. ohiense* had precipitation of driest quarter (36% contribution), mean temperature of warmest quarter (15% contribution), annual precipitation (12% contribution), and minimum temperature of coldest month (10% contribution) as the top climate variables that contributed to model performance (AUC = 0.975). The best-performing model for *H. mississippiense* had mean temperature of coldest quarter (51% contribution), precipitation of coldest quarter (15% contribution), and mean temperature of the warmest quarter (12% contribution) as the top climate variables that contributed to model performance (AUC = 0.883). Environmental modelling also suggested that niche overlap between the two species of *Histoplasma* in North America might be larger than our collections suggest as they might co-occur in Missouri, Arkansas, Louisiana, Mississippi, Alabama, Georgia, Tennessee, Kentucky, North Carolina, South Carolina, Illinois, Indiana, Virginia and Ohio. Figure 4 shows a scenario inferred with 75% probability.

## Discussion

Histoplasmosis, an endemic fungal disease in the US, is primarily reported in midwestern, eastern, and central states. However, the comprehensive understanding of its epidemiology in the US remains incomplete due to limited notifiable cases, inadequate diagnostics and awareness in non-endemic regions, and a poorly described ecological niche [12, 39]. Nonetheless, there are indications that the range of *Histoplasma* in North America is larger than currently thought. *Histoplasma* has been collected outside of the commonly recognized endemic areas, usually from soil and guano, suggesting the fungus might occur in its saprotrophic phase across a larger geographic area [40]. Indeed, our environmental mapping suggests that the environmental conditions propitious for the occurrence of both species *Histoplasma* are broader. These results are not surprising as *Histoplasma* can survive in soil temperatures ranging from −18°C to 37°C [1], but they do suggest that a systematic environmental survey is sorely needed to determine what is the disease's prevalence, identify high-risk areas, and better understand the environmental factors influencing its occurrence.

Our results show that *H. ohiense* is more prevalent in the eastern USA while *H. mississippiense* is commonly found in the western region of the USA (Figure 3). Notably, the predicted geographic ranges for the two species in North America are mostly governed by different environmental conditions. Precipitation of driest quarter and mean temperature of coldest quarter are the most important variables for *H. ohiense* and *H. mississippiense*, respectively. This is in agreement with yeast growth curves performed at 37°C; *H. ohiense* has optimal density growth higher than *H. mississippiense* [21].

Both the phylogenetic and PCA analysis reveal two distinct genotypes within *H. mississippiense*: Clade I and Clade II. Clade I predominantly comprises veterinarian samples recovered mostly in Oklahoma, while clade II consists exclusively of clinical samples recovered mostly in Missouri. Notably, both cats and dogs exhibit higher average body temperatures than humans (38°C-39°C). Consequently, representatives of *H. mississippiense* Clade I may have acquired unique thermotolerance traits facilitating their survival in pets. However, comprehensive thermotolerance assays, including different genotypes within and between species, are imperative to ascertain whether this clade exhibits genotype-specific thermo-adaptation traits. The clustering of a significant proportion of Clade 1 strains from dogs and cats prompts an intriguing consideration: Could this clade possess an enhanced adaptation to higher body temperatures typically observed in these animals, which are 1–1.5°C higher than humans? Since the ability to transition to yeast is a key trait in virulence (reviewed in [41]), the influence of temperature on the distribution of these two pathogens deserves a more systematic treatment.

A second result from the ENM is that the geographic range of the two species overlaps across the Midwest (Figure 4). This geographic overlap is of importance because the two species have been reported to have exchanged alleles recently [42]. Additionally, our discovery of a balanced distribution of *MAT1-1*/*MAT1-2* idiomorphs aligns with expectations for sexually recombining species, corroborating earlier findings in *Histoplasma* within the US [43]. This poses the question to whether there is a tension zone in which the two species not only overlap but also hybridize, which in turn might act as a source of variation for each of the two species. Hybridization in fungi seems to be prevalent and has been proposed to be an important evolutionary mechanism that can generate new trait combinations in admixed individuals. Genomic studies have revealed that indeed introgression is common across fungal species boundaries [20,44,45]. Despite the potential importance of hybridization in fungi, few hybrid zones have been identified [46,47]. A more systematic environmental sampling across the US in *Histoplasma* is needed to determine whether hybridization shows a geographic structure.

An additional finding from our sampling was the two isolates of *H. suramericanum* found in North America. Two potential non-mutually exclusive explanations can illuminate this observation. First, *H. suramericanum* might be endemic in the US. The isolation of a clinical sample from this species in Texas suggests that this species might be endemic to Southern USA but it does not provide evidence for or against the idea that *H. suramericanum* may contribute to autochthonous cases of histoplasmosis in the country. The second possibility is that patients from other locations in the species’ range might have migrated to the USA. The isolate B10265 was recovered from a patient from Richmond, California, with unknown travel history, and we can’t infer the precise location of infection. It's worth mentioning that the metadata associated with those isolates is old and future work is needed to infer the distribution of *H. suramericanum* in North America. In addition, this species has also been found to be autochthonous in Alberta and Montreal, Canada [17], which in turn suggests it might have a much larger range than previously thought. The areas of overlap of different species of *Histoplasma* seem to be extensive, and even though they might have the opportunity for regular gene exchange, introgression is limited.

One factor that might affect species distribution of the different *Histoplasma* species is the geographic range of their potential hosts [15]. Bats, and birds to a certain extent, are considered to be key for the dispersion of *Histoplasma* [5,15,48,49]. Isolation of *Histoplasma* from over six different species of bats (guano or internal organs), belonging to different genera, suggests that bats are a natural host of the fungus across the Americas [50,51,52], Europe [53], Africa [54] and Asia [55]. Bats are a widely distributed and diverse group [56], posing the question of whether there has been host-fungus coevolution and subsequent specialization. Most of the bat surveys predate genomic and metagenomic surveys. Our study focuses on the importance of abiotic factors to determine the potential niche of two different species of *Histoplasma*. Now that a more complete picture exists of the geographic range of *Histoplasma*, studies assessing the co-occurrence of different species of *Histoplasma* and potential hosts, and even potential competitors (e.g. other species of dimorphic fungi), will make it possible to determine the importance of biotic factors in the geographic range of *Histoplasma*.

Nevertheless, it is imperative to recognize some limitations in our study. The uneven distribution of isolates among states may result in an incomplete representation of the fungus's geographic prevalence. Additionally, a significant proportion of clinical samples lacked documented infection events or travel history due to the antiquity of the samples, leading to data gaps. Moreover, the absence of standardized medical records and reports hampers a comprehensive analysis of differences of histoplasmosis clinical manifestations among the examined clinical strains from both species. The study is based on the analysis of 93 *Histoplasma* isolates, including 62 novel genomes, which may not fully represent the thousands of infections occurring annually in the United States. This limitation also applies to the ecological niche modelling (ENM) analysis, which relied on a limited number of genotyped isolates. Therefore, comprehensive collections through epidemiological survey studies are essential to address these gaps. Despite the sampling limitations, our study confirmed the presence of the two major genotypes, NAm1 and NAm2, as the primary causative agents of histoplasmosis. We also observed their prevalence in both suggested endemic areas and non-endemic states. Furthermore, *H. mississippiense* exhibited higher genome-wide nucleotide diversity (π = 0.57%) than *H. ohiense* (π = 0.14%), and the respective PCA plots corroborated this pattern. While the PCA analysis for *H*. mississippiense revealed minor variance, it supported the Structure analysis, indicating two distinct populations within that species: *H. mississippiense* clades I and II. Conversely, *H. ohiense* did not display a similar population structure in the PCA plot, suggesting the need for more isolates to investigate the possibility of new genotypes within this clade.

In this study, we combined whole-genome sequencing, phylogenetics, and species distribution models (SDM) to understand the species distribution of fungal pathogens. Most epidemiological maps are based on pre-1970s data [13]**.** This study successfully demonstrates the integration of a multifaceted approach, along with epidemiological data, to deduce the distribution of fungal pathogens. For example, a recent study on *Coccidioides spp.* distribution modelling successfully achieved a distribution pattern with defined inputs that correlated with areas of high coccidioidomycosis incidence in the USA [57]. Similarly, emerging areas of histoplasmosis were predicted using a modelling approach [12,58]. By using molecular epidemiology and whole-genome sequence typing, a fuller understanding of the current epidemiological scenario and species diversity causing histoplasmosis in the US is within reach.

## Supplementary Material

Supplementary_TablesR1

Supplementary_Figure_1
