# Supplementary figures and images for: Tracing histoplasmosis genomic epidemiology and species occurrence across the USA
Source: Emerg Microbes Infect. 2024 Mar 11;13(1):2315960. doi: 10.1080/22221751.2024.2315960 (PMC10930103; doi:10.1080/22221751.2024.2315960)

A

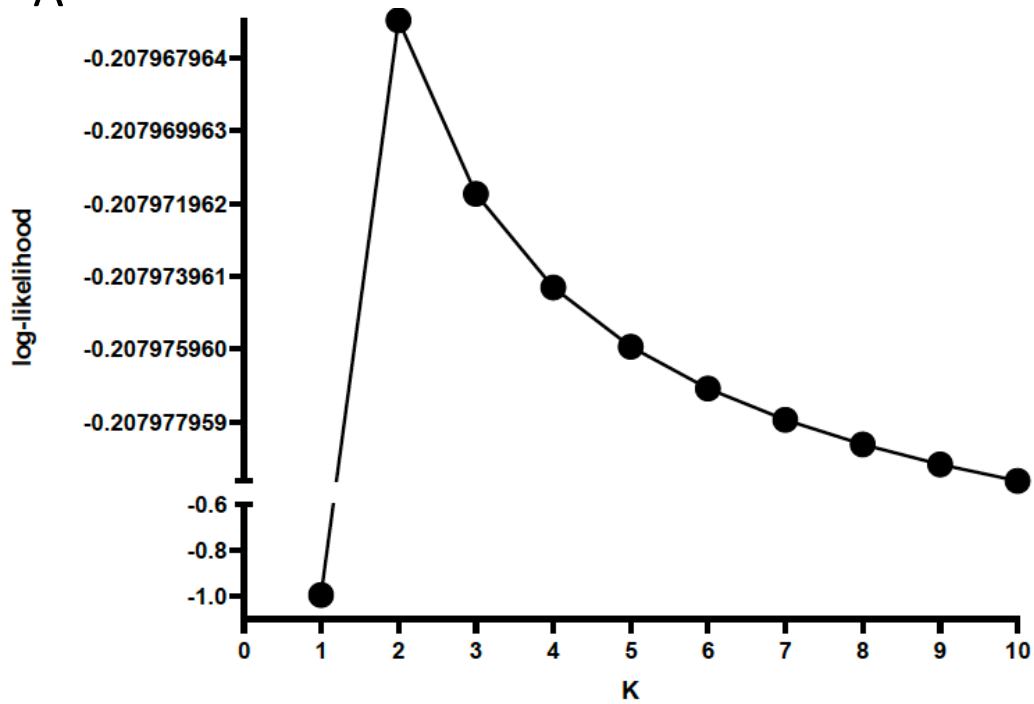

B

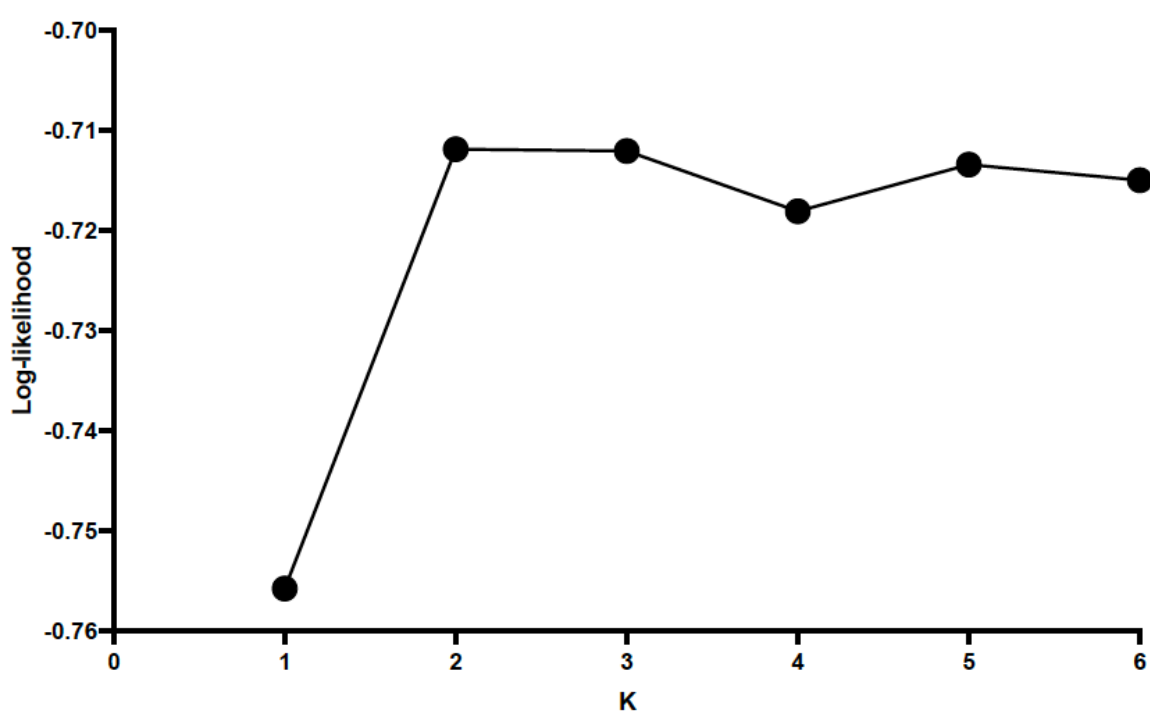

C

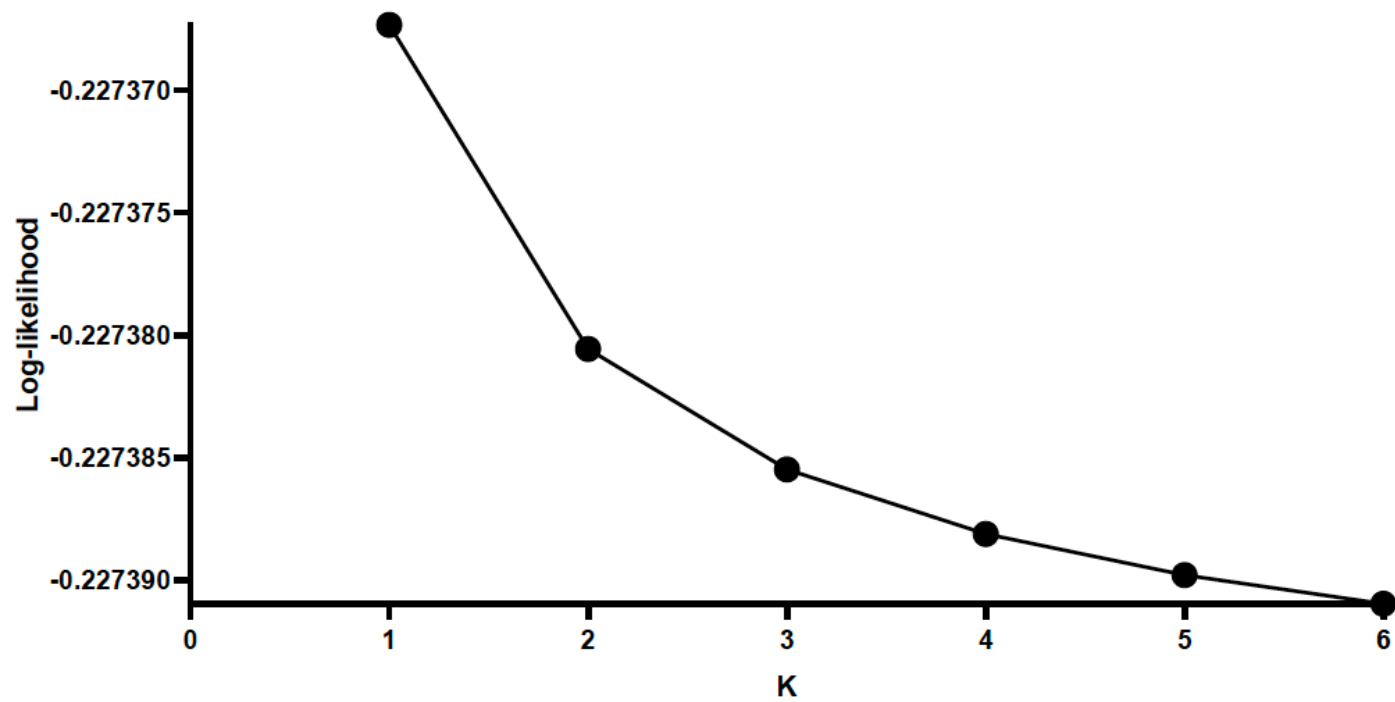

Supplement: Supplementary_Figure_1 [file TEMI_A_2315960_SM9897.pdf]
